# A novel thermostable polymerase for RNA and DNA loop-mediated isothermal amplification (LAMP)

**DOI:** 10.3389/fmicb.2014.00395

**Published:** 2014-08-01

**Authors:** Yogesh Chander, Jim Koelbl, Jamie Puckett, Michael J. Moser, Audrey J. Klingele, Mark R. Liles, Abel Carrias, David A. Mead, Thomas W. Schoenfeld

**Affiliations:** ^1^Lucigen CorporationMiddleton, WI, USA; ^2^Department of Biological Sciences, Auburn UniversityAuburn, AL, USA

**Keywords:** diagnostics, RNA/DNA polymerase, infectious diseases, RT-LAMP, point-of-care

## Abstract

Meeting the goal of providing point of care (POC) tests for molecular detection of pathogens in low resource settings places stringent demands on all aspects of the technology. OmniAmp DNA polymerase (Pol) is a thermostable viral enzyme that enables true POC use in clinics or in the field by overcoming important barriers to isothermal amplification. In this paper, we describe the multiple advantages of OmniAmp Pol as an isothermal amplification enzyme and provide examples of its use in loop-mediated isothermal amplification (LAMP) for pathogen detection. The inherent reverse transcriptase activity of OmniAmp Pol allows single enzyme detection of RNA targets in RT-LAMP. Common methods of nucleic acid amplification are highly susceptible to sample contaminants, necessitating elaborate nucleic acid purification protocols that are incompatible with POC or field use. OmniAmp Pol was found to be less inhibited by whole blood components typical in certain crude sample preparations. Moreover, the thermostability of the enzyme compared to alternative DNA polymerases (*Bst*) and reverse transcriptases allows pretreatment of complete reaction mixes immediately prior to amplification, which facilitates amplification of highly structured genome regions. Compared to *Bst*, OmniAmp Pol has a faster time to result, particularly with more dilute templates. Molecular diagnostics in field settings can be challenging due to the lack of refrigeration. The stability of OmniAmp Pol is compatible with a dry format that enables long term storage at ambient temperatures. A final requirement for field operability is compatibility with either commonly available instruments or, in other cases, a simple, inexpensive, portable detection mode requiring minimal training or power. Detection of amplification products is shown using lateral flow strips and analysis on a real-time PCR instrument. Results of this study show that OmniAmp Pol is ideally suited for low resource molecular detection of pathogens.

## Introduction

Rapid, sensitive, easy-to-use methods for detection of pathogens are needed for timely diagnosis of infectious diseases especially at point-of-care (POC). Common molecular detection methods by end point and real time PCR are valuable tools for pathogen detection and are widely used in clinical diagnostics because of high sensitivity and specificity (Segawa et al., 2014). However, there are several problems in implementing these methods at POC, particularly the need for trained personal, extensive sample preparation protocols and specialized laboratory equipment, which have prevented use of these methods in resource limited settings. Isothermal amplification methods such as loop mediated amplification (LAMP; Notomi et al., 2000) hold great promise to shorten nucleic acid detection times, simplify the instrumentation and reduce power requirements by eliminating the need for thermal cycling. These improvements are facilitating the movement of nucleic acid tests (NATs) from the central laboratory to POC environments like clinics, hospital emergency rooms, farms and other remote areas of need, but still require improvement before fulfilling their potential.

While LAMP has proven highly useful in laboratory environments, the current formats have limited application under POC conditions (Nijru, 2012). Improvements in the DNA polymerase and detection modes could allow use of the LAMP platform in POC testing in resource limited settings. A key drawback of typical LAMP formulations is the inability to directly detect RNA without a second reverse transcriptase enzyme. Currently, most LAMP methods use a truncated product (large fragment) of *Bacillus stearothermophilus* (*Bst*) Pol (Huang et al., 1999) or a highly similar enzyme from closely related moderately thermophilic bacterium. While this enzyme is highly effective for amplification of DNA based targets, it cannot amplify RNA without the addition of a reverse transcriptase for conversion of RNA template to cDNA that serves as a target for LAMP. This adds additional steps and necessitates use of a buffer that is a compromise between the optimal conditions for the respective enzymes. The reaction requirement for the reverse transcriptase also imposes a limit on the thermal stability of the reaction.

Some of the most important RNA targets for diagnostic detection are viral genomes, which can be highly structured. Thermal treatment during sample preparation immediately prior to amplification has been indispensable in allowing direct detection of bacterial and viral targets. Currently available reverse transcriptase's, Avian Myeloblastosis Virus (AMV RT) or Moloney Murine Leukemia Virus (MMLV RT), are relatively labile and thermal melting to alleviate secondary structure has not been possible with any of the current LAMP systems. While single enzyme RT-LAMP methods are available (http://www.optigene.co.uk/), most RT LAMP uses the two-enzyme format.

In order to use NATs in POC settings, it is important to have a simple and easy to use method for detecting amplification. Ideally the detection would confer additional specificity and sensitivity, while keeping total testing costs low. Most common detection methods for LAMP, such as agarose gel electrophoresis or use of real-time PCR instruments are prohibitively expensive, slow, and require extensive user training. Dyes such as calcein (Tomita et al., 2008), or hydroxynapthol blue (HNB; Goto et al., 2009) in the LAMP reaction mixture allows direct visual detection of amplification results, but do not improve specificity and the ambiguous results require more user judgment than is acceptable for POC use. An alternative detection mode is the use of lateral flow devices (LFD), which can be portable, and does not require instrumentation or electrical power. The combination of LAMP and LFD provides an inexpensive, facile tool for NAT in remote, low resource environments. The need for a refrigerated cold chain is unavailable in many low resource settings, which impairs the utility of a POC test, so a final component of a molecular based POC technology is the stability of the test for distribution and storage under ambient conditions.

Screening viral metagenomes from boiling hot springs uncovered new thermostable DNA polymerases (Schoenfeld et al., 2008). An engineered derivative of one of these, PyroPhage 3173 DNA polymerase, was effective in RT PCR (Moser et al., 2012). This enzyme exhibits innate reverse transcriptase activity, thermostability and potent strand-displacing activity and has now been formulated for use in direct detection of RNA and DNA pathogens by LAMP. Its thermostability allows additional flexibility for using a thermal treatment in sample preparation and amplification of highly structured regions of genomes.

In this report we describe the use of this novel polymerase in LAMP and RT-LAMP (reverse transcription LAMP). In order to understand the potential applications and limitations of using OmniAmp polymerase in LAMP, a diverse group of DNA and RNA based targets were selected (Table 1). In addition to developing LAMP method for each pathogen, we also evaluated the use of a lateral flow device to detect the amplification results and validated the use of dried reagents stable to ambient storage as a step in providing POC LAMP assays.

**Table 1:** **List of targets for which LAMP assays were developed using OmniAmp polymerase**.

**Pathogen**	**Genome**	**Target gene**	**Incubation temp. (°C)**	**Source**
MS2 phage (MS-2)	RNA	Replicase protein (MS2g4)	70	ATCC
Swine influenza virus (SIV) H1N1	RNA	Matrix (M)	72	University of Minnesota, St. Paul, MN
Porcine circovirus-2 (PCV-2)	DNA	Capsid protein (ORF 2)	70	
West Nile virus (WNV)—NY 2001-6263	RNA	Envelope glycoprotein	72	ZeptoMetrix, Buffalo, NY
*Edwardsiella ictaluri*	DNA	Repetitive element	70	Auburn University, AL
*Bacillus atrophaeus* (BAT)	DNA	ATP synthase, β-subunit	68	Steris Corporation, OH
*Staphylococcus aureus* MSSA	DNA	Carbamate kinase (*arc*C)	68	ZeptoMetrix, Buffalo, NY
^*^Ebola virus (EBoV)—Zaire	RNA	Glycoprotein (GP)	72	Galveston National Lab, TX (RNA only)
^*^Crimean-Congo hemorrhagic fever virus (CCHFV)	RNA	Nucleoprotein (S)	68	
Bovine viral diarrhea virus (BVDV)—type I	RNA	5′-UTR	70	Wisconsin Veterinary Diagnostic Laboratory, Madison, WI

* *RNA extracts were provided by Galveston National Laboratory, TX and were certified for use in BSL II facility*.

## Materials and methods

### LAMP enzymes

The discovery and initial characterization of PyroPhage 3173 DNA polymerase and its application in RT-PCR has been described earlier by Schoenfeld et al. (2008) and Moser et al. (2012). The wild type DNA polymerase had a potent proofreading exonuclease activity that was disabled by mutagenesis. The modified enzyme was formulated for use in LAMP and RT LAMP and is commercially available as OmniAmp polymerase (Lucigen Corporation, Middleton, WI). *Bst* DNA polymerase (Lucigen, Corporation, WI) was used to compare DNA LAMP assay results with OmniAmp polymerase.

### Pathogens

Table 1 lists the pathogens for which LAMP assays were developed. All pathogens were obtained from different sources and nucleic acids (DNA or RNA) were extracted from overnight grown cultures (for bacteria) or from cell culture supernatants (for viruses) using commercial kits (Qiagen, Valencia, CA). For Ebola virus (EBoV) and Crimean-Congo hemorrhagic virus (CCHFV), agents of viral hemorrhagic fever, RNA was extracted in a BSL-4 facility at Galveston National Laboratory, TX and tested for safety for use in BSL-II laboratory before being transferred to Lucigen.

### LAMP primer design

For each pathogen, LAMP primers targeting conserved regions of the indicated pathogens were designed using the online primer design utility, Primer Explorer (https://primerexplorer.jp/e/). Conserved regions for the targeted genes were identified by aligning the nucleotide sequences of target genes from GenBank (www.ncbi.nlm.nih.gov) together using clustal W (www.megasoftware.net). Nucleotide sequences (200–300 bp) of the conserved regions as determined by alignment were used to design LAMP primers. Primer designs were selected to provide 100% specificity based on analysis by BLAST (www.ncbi.nlm.nih.gov) and the list of primers is provided in Table 2.

**Table 2:** **List of LAMP primers used in this report**.

**Target**	**Primer**	**Sequence (5′–3′)**	**Reference**
MS2 phage (MS-2)	F3	TGTCATGGGATCCGGATGTT	This study
	B3	CAATAGAGCCGCTCTCAGAG	
	FL	CCAGAGAGGAGGTTGCCAA	
	BL	TGCAGGATGCAGCGCCTTA	
	FIP	GCCCAAACAACGACGATCGGTAAAACCAGCATCCGTAGCCT	
	BIP	GCACGTTCTCCAACGGTGCTGGTTGCTTGTTCAGCGAACT	
Swine influenza virus (SIV) H1N1	F3	ATCATCCCGTCAGGCCCCCTCA	This study
	B3	TACGCTGCAGTCCTCGCTCACTGG	
	FL	TGTCTTTGCAGGAAAGAAC	
	BL	TCTGACTAAGGGAATTTTAGGAT	
	FIP	CCATGAGAGCCTCAAGATCAAGCCGAGATCGCACAGA	
	BIP	GACAAGACCAATCCTGTCACCACGGTGAGCGTGAACACAA	
Porcine circovirus-2 (PCV-2)	F3	CACTTCGTAATGGTTTTTATTATTTA	Zhao et al., 2011
	B3	TCCACTATTGATTACTTCCAAC	
	FL	AACCATGTATGTACAATTCAGAGAATTTAATC	
	BL	TTCCAGCAGTTTGTAGTCTCAGC	
	FIP	CAGGAATACAATATCCGTGTAACCATTTTGGTTAAGTGGGGGGTCTT	
	BIP	GAGGCCTACGTGGTCTACATTTTTCAAACAACAAAAGAAATCAGCTATG	
West Nile virus (WNV)—NY 2001-6263	F3	TGGATTTGGTTCTCGAAGG	This study
	B3	GGTCAGCACGTTTGTCATT	
	FL	CATCGATGGTAGGCTTGTC	
	BL	TCTCCACCAAAGCTGCGT	
	FIP	TTGGCCGCCTCCATATTCATCATTTTCAGCTGCGTGACTATCATGT	
	BIP	TGCTATTTGGCTACCGTCAGCGTTTTTGAGCTTCTCCCATGGTCG	
*Edwardsiella ictaluri*	F3	CGGCGAAAATCATACCCCT	This study
	B3	ACCCGACAGACAGAGGAAAG	
	FL	GGCAAGAGAGGACGACCACGATA	
	BL	CAGAGACAAGCACGGCGAGTG	
	FIP	ATTGTTGGATGCCCTCCCGGGTCTGCGTGTAGCTTGTCA	
	BIP	TCGAGTCATGGCGATTGGCTCCGACACATAGTGGTGGAACG	
*Bacillus atrophaeus* (BAT)	F3	GTCGCCTAAATGAAGTGC	This study
	B3	GGATAGCGATGAAGAAAGGAC	
	FL	AGGTGAAAATGAAGTAGG	
	BL	AAAACGTACGTCAACGA	
	FIP	CGATTAAAGTTTCACAACCAGCAACGACCTCTAGCGTTAAATCGA	
	BIP	ATTTCAGGTAAGTGACCGTCTTCCGTTAGCCAAGTAATGGGAC	
*Staphylococcus aureus* MSSA	F3	TCGAACAGTGACACAACG	This study
	B3	TCTTCTTTCGTATAAAAAGGACC	
	FL	CCTATCATACCCTGTGACATT	
	BL	ACACGTGTGGAAGTAGATAA	
	FIP	GCGATTGATTTCAGTTTCCAACCCATTGGATACTTGTGGTGC	
	BIP	AGTGATAGAACTGTAGGCACAATCGTTATCAAATCGTGGATCATCT	
Ebola virus—Zaire	F3	ATGGGCTGAAAACTGCTACA	This study
	B3	CAGCGAAAGTCGTTCCTCG	
	FL	GTCTGGCGCTGCTGGTAGAC	
	BL	CCTTCCACAAAGAGGGTG	
	FIP	TTGTGCACATACCGGCACCGAAAAAACCTGACGGGAGTGA	
	BIP	GACCGTGTGCCGGAGACTTTGTGGAAGCAAGTCGATCAT	
Crimean-Congo hemorrhagic fever virus (CCHFV)	F3	AGGTGGTTTGAAGAGTTCA	This study
	B3	ACAAAACTTTGTTGCCTCC	
	FL	ATAGGAGTTTGTGAAGGTGT	
	BL	CCGATGATGCACAGAAGG	
	FIP	TGGGAACACTCTCGCAAAAGGAAAAAGGAAATGGACTTGTGG	
	BIP	TGTGTTTCAGATGGCCAGTGCCGAGCAGATGCGTAGATGGAG	
Bovine viral diarrhea virus (BVDV)—type I	F3	GCGAAGGCCGAAIAGAGG	Koelbl et al., in preparation
	B3	TITGGGCITGCCCTCG	
	BL	CAGGGTAGTCGTCAGTGGTTC	
	FIP	CICCACTGITGCTACCCICCTAICCATGCCCTTAGTAGG	
	BIP	CGTTGGATGGCTIAAGCCCTGAGTCCACITGGCATCTCG	

For use in LAMP reaction, 20X primer mix was prepared by mixing all six primers (F3,B3:FL,BL:FIP,BIP) in 1:4:8 ratio (Nagamine et al., 2002). Primer mix was stored at −20°C till used.

### Optimization of LAMP assay

LAMP assays were developed using OmniAmp 2X Isothermal Master Mix (Lucigen Corporation, WI). This master mix is formulated for LAMP and contains optimal concentrations of betaine, salts, dNTPs, and OmniAmp polymerase. Reactions were formulated and performed as described in Lucigen's OmniAmp manual. Final concentration of the reaction mixes were: 1X OmniAmp Master Mix, 2 mM Fiona Green dye (Marker Gene, OR), and 1X LAMP primer mix (IDT, IA; stock solution: 20X); 5 μl of target (DNA or RNA), brought to volume (25 μl) with DNase-RNase free water and incubated in a real time thermocycler (iQ5, Bio-Rad, CA) at constant temperature for indicated times and monitored by detection of Fiona Green fluorescence, measured and quantified by the instrument software at 30 s intervals. The TTR (time to result) was set as the time at which the fluorescence crossed a hypothetical threshold of 10% of maximal fluorescence. Samples were considered negative if they failed to cross the threshold. In each case, at least three primer sets were synthesized and compared for TTR and specificity. Post-amplification melt analysis was used to distinguish correct (target-dependent) from spurious (target-independent) amplification products. To further verify specificity, reaction products were also visualized by electrophoresis on ethidium bromide-stained 2% agarose gels. Optimal amplification temperatures for each assay were determined using a temperature gradient ranging from 66 to 74°C. To determine the sensitivity of assay, 10-fold serial dilutions of DNA or RNA was prepared in water for detection by LAMP.

### Development of rapid sample preparation method

We also evaluated use of a simple heat lysis method for the extraction of nucleic acid from different clinical matrices. Heat lysis was performed by diluting sample into an extraction buffer followed by incubation at 90°C for 5 min. After incubation, lysates were used as template in LAMP reaction as described in above section Optimization of LAMP Assay.

For this, sheep whole blood (Hemostat Laboratories, CA) was spiked with *E. coli* MS2 RNA virus particles followed by 10-fold serial dilutions in the same matrix. As a control, 10-fold dilutions of virus particles were made in Tris buffer. Spiked samples were divided into two parts, one part was extracted using a heat lysis method and the other part was used for viral nucleic acid extraction using a commercial kit (QIAamp Viral RNA extraction kit, Qiagen, CA). For heat lysis, samples were diluted in a Tris-EDTA extraction buffer (Lucigen Corporation, WI) and incubated at 90°C for 5 min. After extraction, lysates from both methods were used directly as template in LAMP.

### LAMP with lyophilized reagents

To allow ambient storage of formulated LAMP reagents, 1X isothermal master mix, including OmniAmp polymerase was prepared without glycerol, primers, and Fiona green dye. LAMP formulation was lyophilized using BioLyph's (Hopkins, MN) patented technology. Lyophilized LAMP reactions were rehydrated with template, primers and dye into a total volume of 25 μl and incubated and detected in a real time thermocycler run isothermally as described above.

### Detection of amplification by using lateral flow device

To simplify detection of positive reactions, we evaluated use of LFD. For this application, forward and reverse loop primers were synthesized with a 5′-conjugated biotin and FITC, respectively. The LF strips were prepared in-house (Lucigen Corporation, Middleton, WI) using an anti-biotin antibody (Thermo Scientific, IL) for capture and a colloidal gold-conjugated anti-FITC antibody (British Biocell International, UK) for detection. In this application LAMP was performed as described above using labeled loop primers and after completion, reaction products were loaded on the LFD for detection. A positive reaction was indicated by the appearance of red lines at both “Control” and “Test” whereas the appearance of a red line only at “Control” indicates a negative reaction.

This method was evaluated using two strains of *Edwardsiella ictaluri* (S97-9773 and 219). Specificity was determined using one strain each of *Edwardsiella tarda* and *Escherichia coli* (DH10B). For LAMP, six 100-fold dilutions (−2, −4, −6, −8, −10, and −12) of each strain were made in Tryptic Soy Broth (TSB) from overnight grown cultures. These dilutions were used directly as template in *E. ictaluri* LAMP assays. After incubation, LAMP reaction products were loaded on to LFD for visualization.

## Results

### Comparison of OmniAmp and *Bst* polymerases

**Figure 1:**
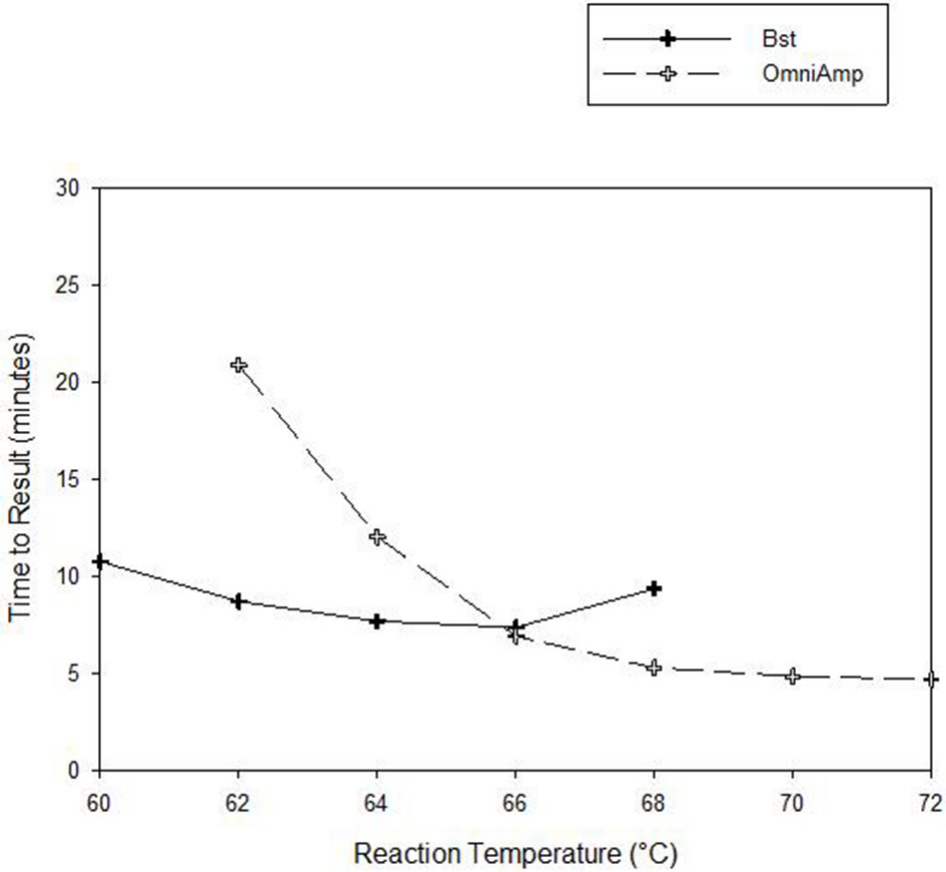
**Performance as measured by time to result of OmniAmp and *Bst* polymerases at varied reaction temperatures in detecting *Edwardsiella ictaluri* by LAMP**.

**Figure 2:**
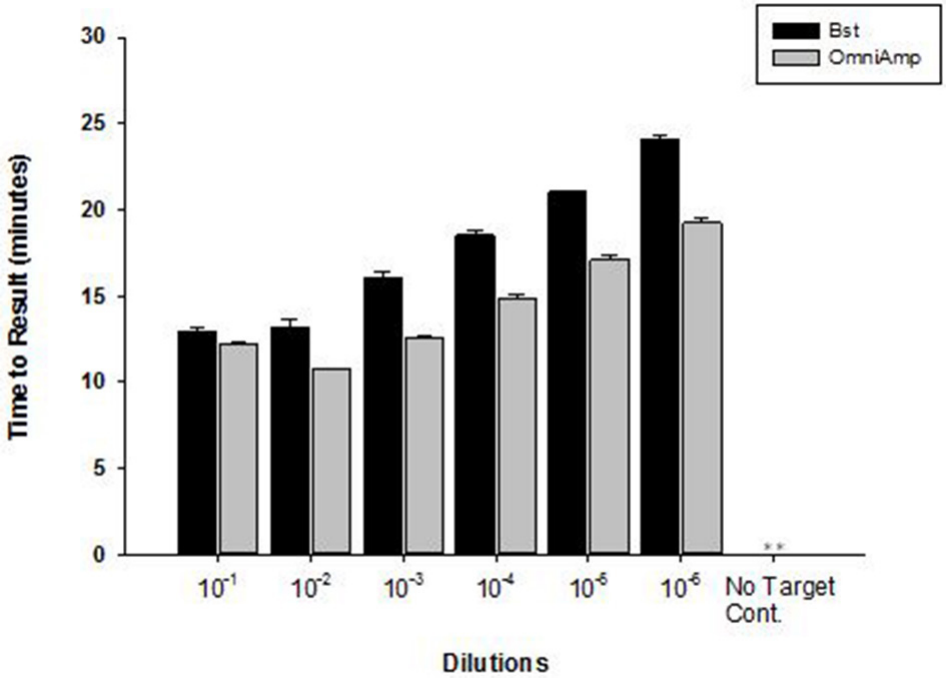
**Performance of *Bst* and OmniAmp polymerases as measured by time to result in *Edwardsiella ictaluri* LAMP**. *Bst* and OmniAmp LAMP were performed at their optimal temperatures, 65 and 70°C, respectively. (No amplification is indicated by “^**^”).

Performance of OmniAmp polymerase in a LAMP reaction was compared with that of *Bst* polymerase (Figure 1). The temperature optimum of OmniAmp is about 70°C, while that of *Bst* is 65°C. At its optimal temperature, the OmniAmp polymerase was significantly faster than *Bst* polymerase. This translates to a shorter time to result (TTR), as shown in the detection of the DNA target in *Edwardsiella ictaluri*, an important catfish pathogen. This advantage in shorter TTR was more pronounced at lower template concentrations where detection by the OmniAmp polymerase was 20% faster (Figure 2).

### Detection of DNA targets

**Figure 3:**
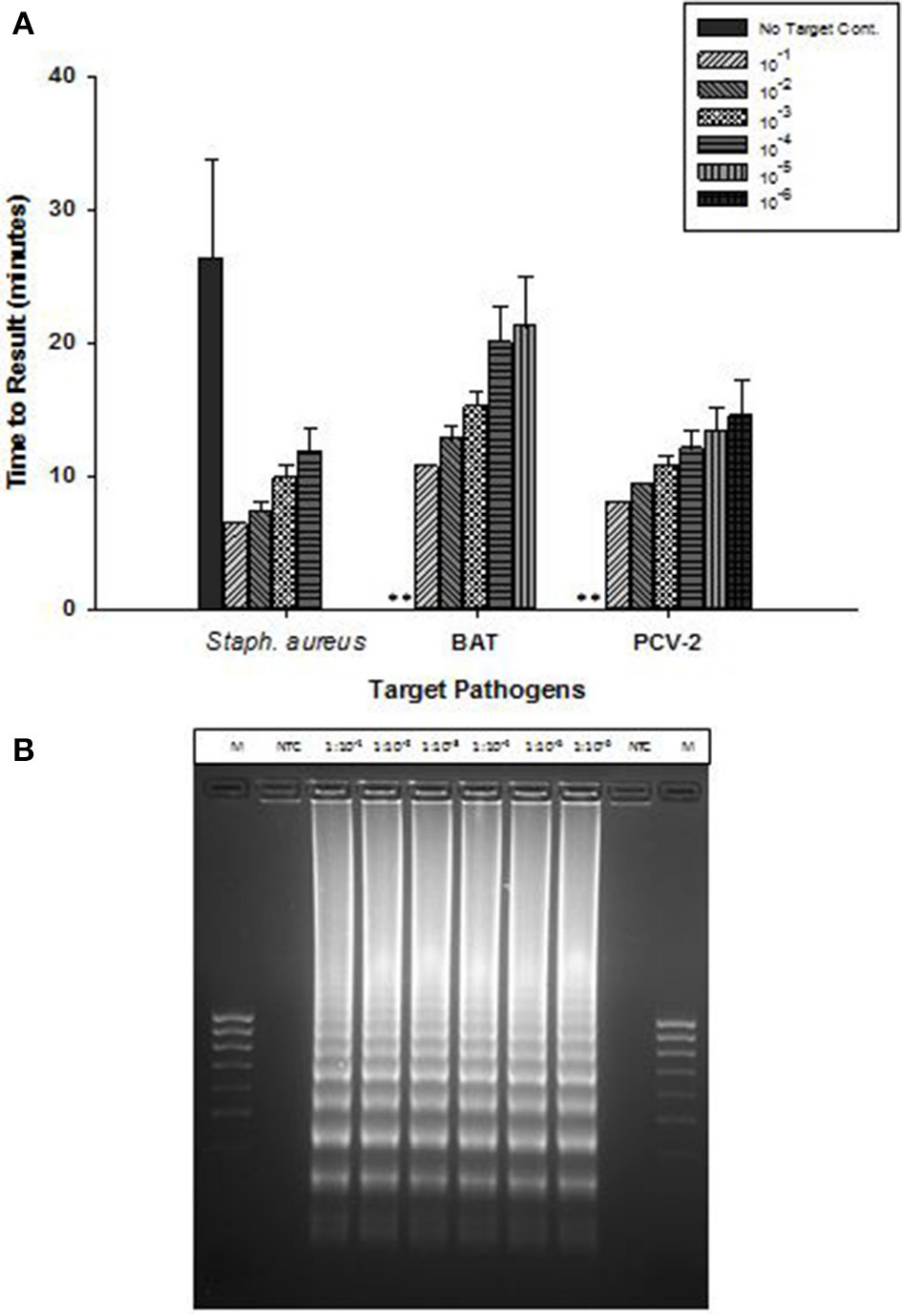
**Detection of DNA targets: *Staphylococcus aureus* (*Staph. aureus*), *Bacillus atrophaeus* (BAT), and Porcine circovirus (PCV-2) using OmniAmp LAMP assays. (A)** For each pathogen, 10-fold serial dilutions of extracted DNA were prepared in water and used as template in LAMP reaction. Amplification was performed on a real time thermocycler in triplicate with average TTRs shown for each dilution. (No amplification is indicated by “^**^”). **(B)** PCV-2 LAMP products were separated on 2% agarose gel.

OmniAmp Pol-based LAMP assays were developed for detection of *Staphylococcus aureus*,l *Bacillus atrophaeous* (BAT), and Porcine circovirus (PCV-2), all of which are DNA targets. LAMP primer designs were tested with serial 10-fold dilutions of DNA under optimized reaction conditions. Overall, amplification of all three pathogens was achieved in <30 min with minimal non-specific amplification (Figure 3A). In *S. aureus* LAMP assay, amplification was observed in no-template control (NTC) which was found to be non-specific as it had different melt temperature; melt temperature of specific product: 82.5°C and melt temperature of non-specific product: 84°C (data not shown).

Post-amplification, reaction products were separated on 2% agarose gel and the appearance of ladder like patterns confirmed the correct amplification products. Figure 3B shows PCV-2 LAMP reaction products on 2% gel.

**Figure 4:**
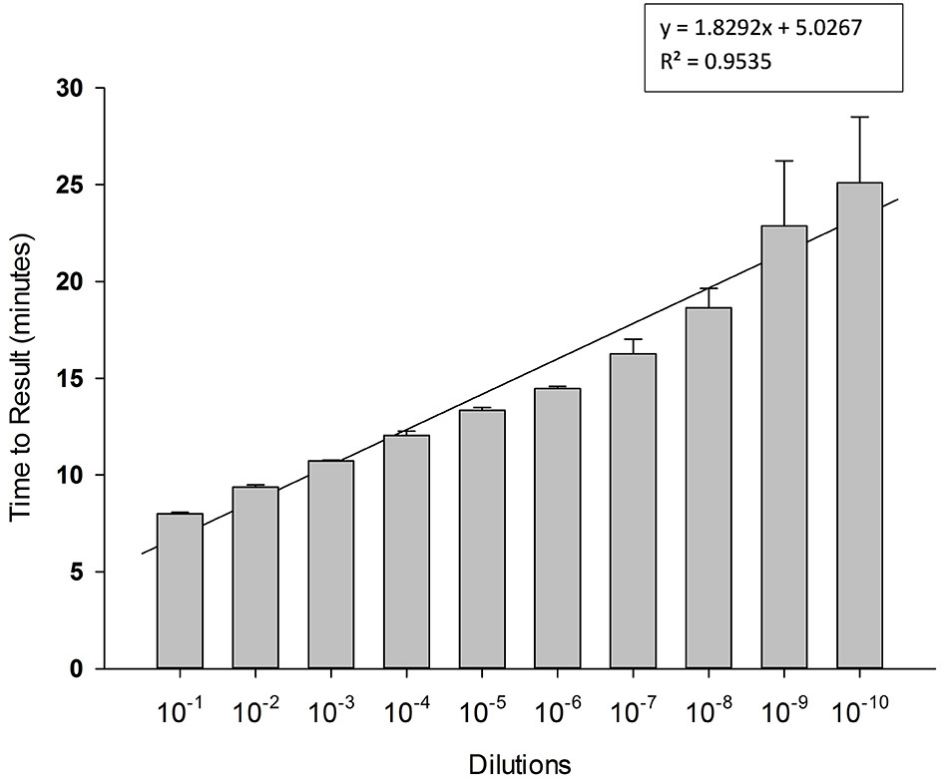
**Sensitivity of Porcine circovirus-2 (PCV-2) LAMP**. 10-fold serial dilutions of extracted DNA were prepared in water and used as template in LAMP reaction. Amplification was performed on a real time thermocycler in triplicate with average TTRs shown for each dilution.

To determine limit of detection, 10-fold serial dilutions of PCV-2 DNA were prepared in water and each dilution was tested in triplicate in LAMP reaction. Results presented in Figure 4 shows high sensitivity of LAMP assay for detection of PCV-2 with limit of detection of about 4 copies of DNA μl. Regression analysis showed good correlation (*R*^2^ = 0.95) between dilutions and time to result (minutes). No amplification signal was detected in any of the negative (no template) controls.

### Detection of RNA targets

**Figure 5:**
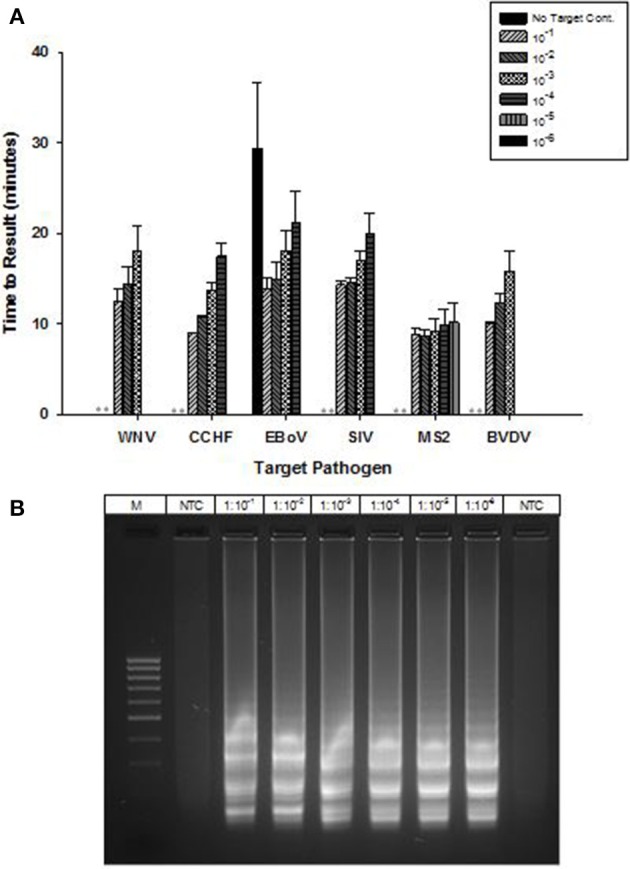
**RT-LAMP using OmniAmp polymerase for detection of RNA targets: West Nile virus (WNV), Crimean-Congo hemorrhagic virus (CCHF), Ebola virus (EBoV), Swine influenza virus (SIV), MS2, and Bovine viral diarrhea virus (BVDV). (A)** For each pathogen, 10-fold serial dilutions of extracted RNA were prepared in water and used as template in LAMP reaction. Results are averages of three TTR values for each dilution. (No amplification is indicated by “^**^”). **(B)** MS2 LAMP products were separated on 2% agarose gel.

OmniAmp Pol has inherent reverse transcriptase activity which enables RT-LAMP detection of RNA targets without modification of the formulation used for DNA targets. RT-LAMP assays were developed for six viruses with RNA genomes (Table 1). Results indicate detection in less than 30 min with no additional steps or modification of the reaction formulations (Figure 5A). After LAMP, reaction products were separated on 2% agarose gel and appearance of ladder like patterns confirmed the correct amplification product as shown in Figure 5B for MS2.

**Figure 6:**
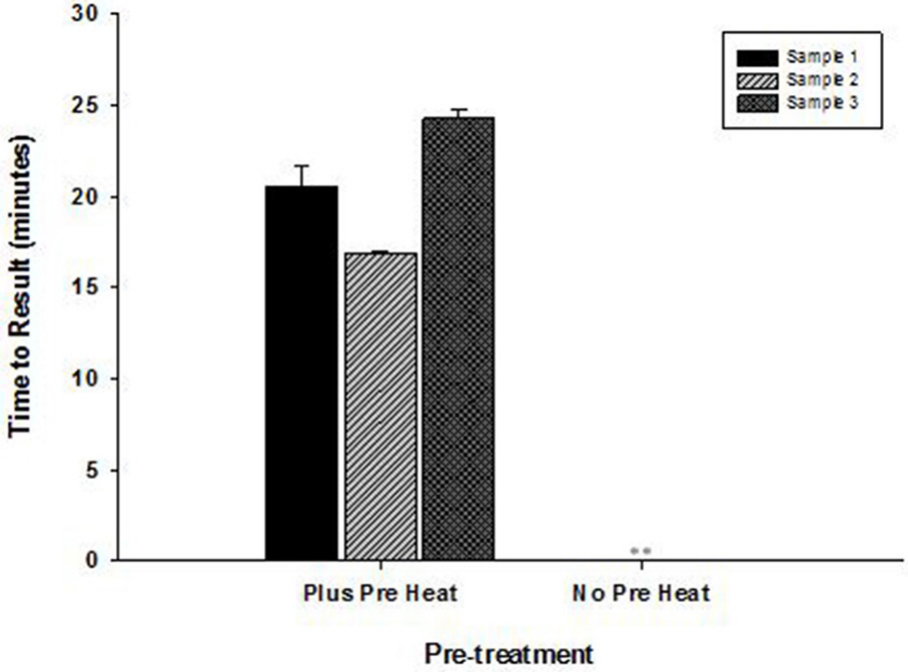
**Effect of pre-incubation step on performance of RT-LAMP for detection of Bovine viral diarrhea virus (BVDV)—type 1**. RNA extracted from three clinical samples (ear notch) were tested in LAMP with pre-heat step (3 s at 92°C) and without pre-heat step before isothermal incubation at 70°C for 30 min. (No amplification is indicated by “^**^”).

High temperature optimum for OmniAmp was found to be indispensable in detecting certain highly structured regions of genomes especially common in RNA viruses. This advantage of OmniAmp was evaluated in developing a LAMP method for detection of BVDV type 1. Primers designed to target the 5′-UTR failed to amplify when used with standard isothermal incubation at 70°C (not shown). A brief incubation at 92°C for 3 s immediately prior to isothermal incubation allowed efficient amplification (Figure 6).

### Rapid sample preparation method

**Figure 7:**
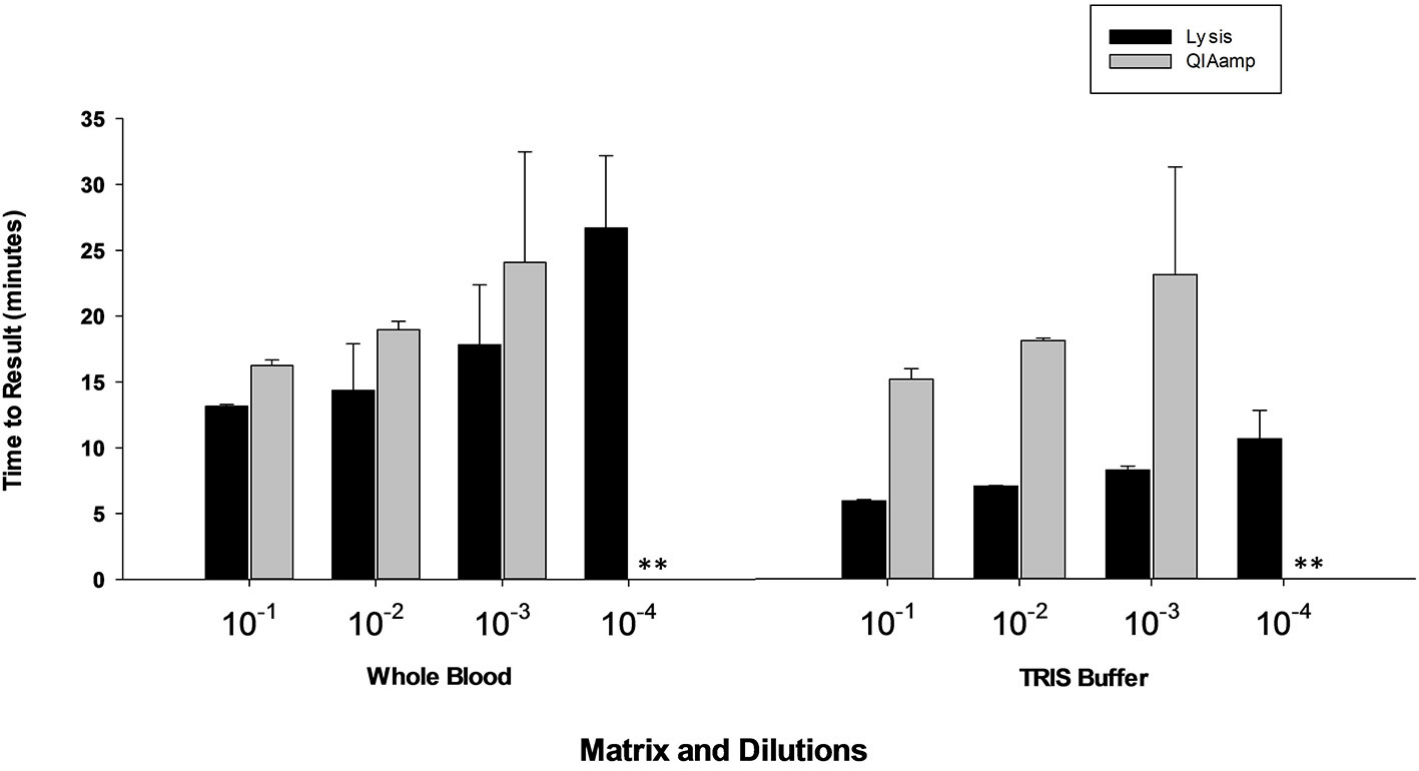
**Feasibility of rapid heat lysis method for extraction of viral (MS2) nucleic acid from blood samples**. Viral RNA from spiked samples was extracted by two methods: rapid heat lysis (Lucigen) and using a commercial RNA extraction kit (QIAamp, Qiagen). (No amplification is indicated by “^**^”).

Results presented in Figure 7 shows that performance of heat lysis method in terms of sensitivity was equivalent to the commercial nucleic acid extraction kit. Presence of inhibitors in whole blood had no effect on the performance of OmniAmp Pol although it did increase TTR when compared to control (dilutions in Tris buffer). We tested the same protocol with other matrices (serum and feces) and results were comparable (data not shown).

### LAMP with lyophilized reagents

**Figure 8:**
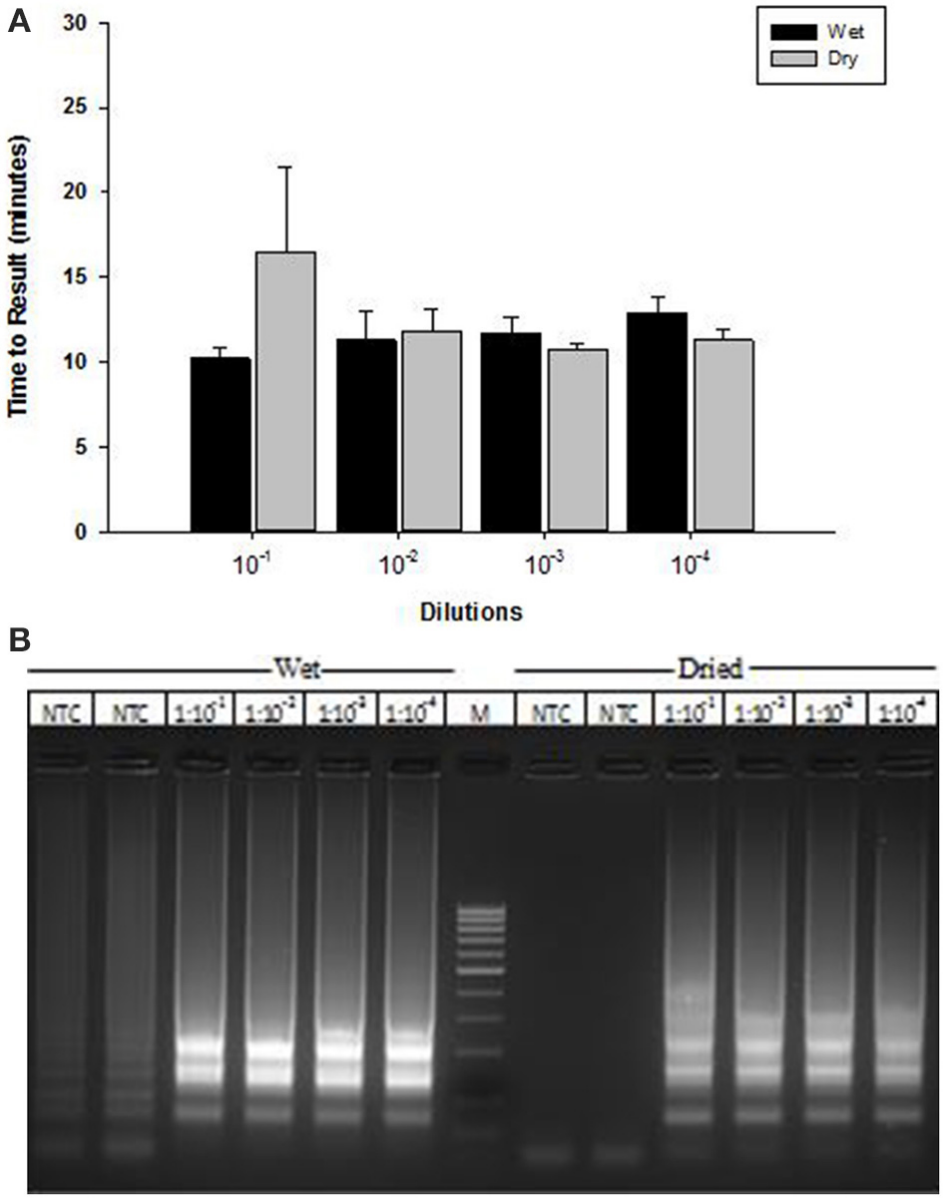
**Comparison of MS2 RT-LAMP with wet and dried reagents. (A)** Ten-fold serial dilutions of MS2 phage particles was made in water and used directly as template in reaction mixture. Amplification was performed on a real time thermocycler in duplicate and time to results was recorded. **(B)** LAMP reaction products were separated on 2% agarose gel.

**Figure 9:**
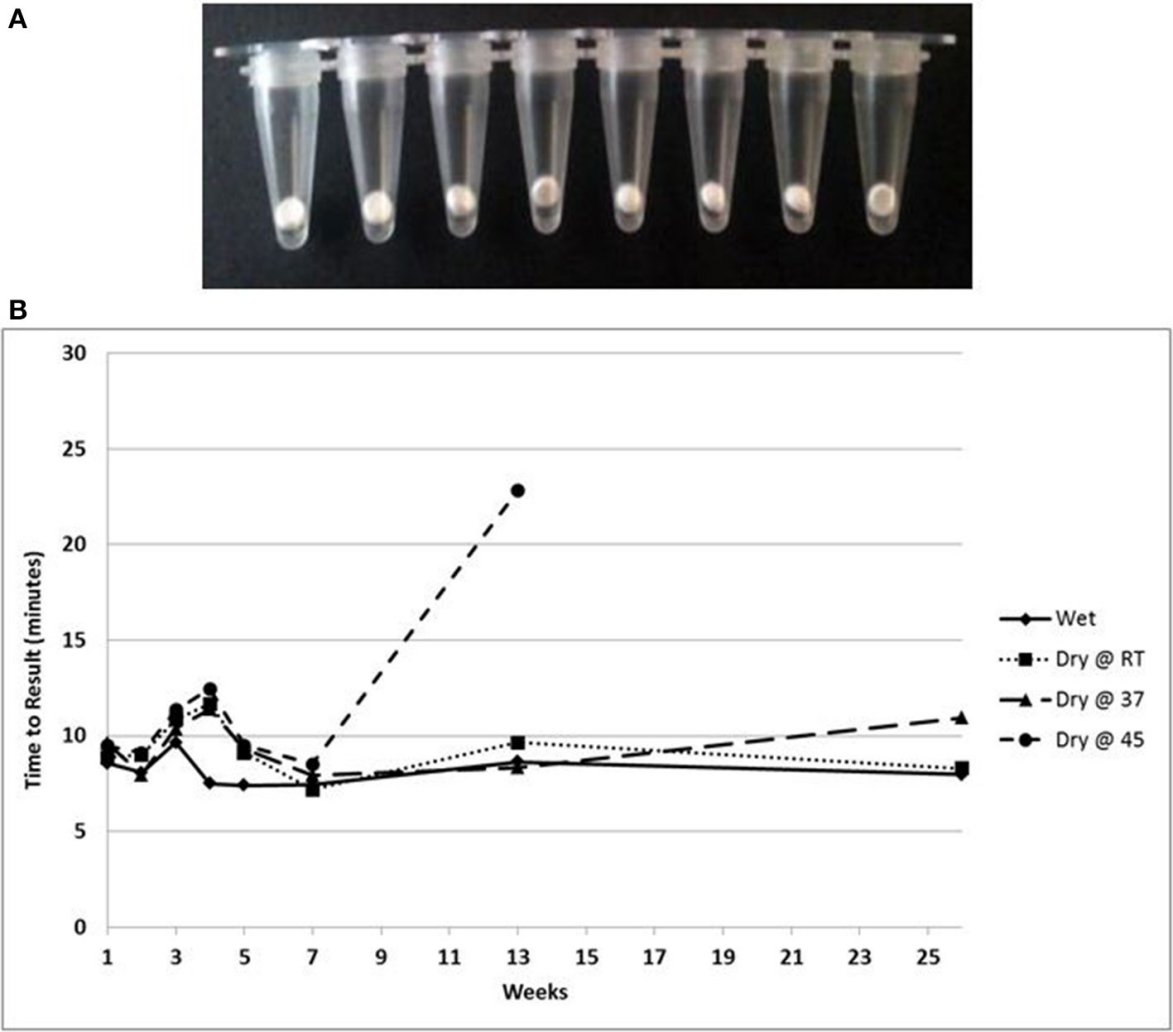
**(A)** Beads of dried LAMP reagents in PCR tubes. **(B)** Feasibility data showing stability based on TTR of LAMP reagents in dried format stored at the indicated temperatures compared to wet enzyme stored at −20° over 180 days.

A lyophilized formulation for the LAMP reagents was compared with wet reagents for detection of MS2 RNA phage target. No diminution of TTR or sensitivity was seen with the dried formulation compared to wet (Figure 8A) nor was an increase in non-specific amplification seen by visualization of LAMP products on a 2% agarose gel (Figure 8B). To evaluate stability, the lyophilized reaction mix (Figure 9A) was incubated at 23, 37, and 45°C and assayed in a *Clostridium difficile* LAMP reaction compared to wet reagent stored at −20°C (Figure 9B). The dried LAMP formulation was stable at 23°C and 37°C for 180 days. The dried reagent was stable at 45°C for 50 days although it did show a measurable drop in TTR after 90 days.

### Lateral flow detection of LAMP products

**Figure 10:**
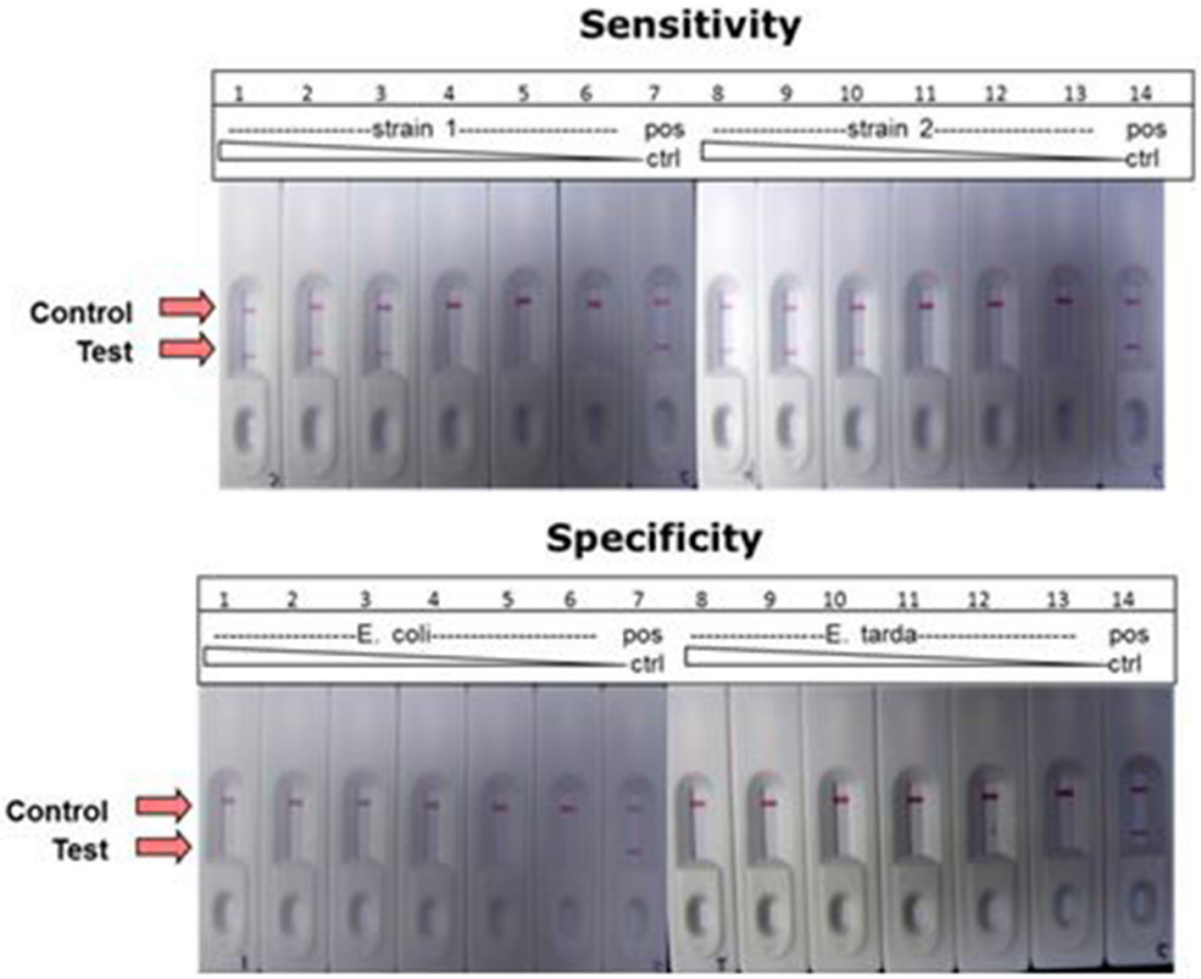
**Detection of *E. ictaluri* LAMP reactions by LFD**. Sensitivity was determined by testing 100-fold dilutions of *E. ictaluri* S97-9773 (strain 1) and *E. ictaluri* 219 (strain 2) in LAMP (**upper panel**). Dilutions over the same range of *E. coli* and *E. tarda* were tested using for the same reactions to confirm specificity (**lower panel**). Positive reaction is indicated by the appearance of two red lines, one at “Control” and other at “Test.” Negative reaction is indicated by appearance of only one red line at “Control.”

A combination of LAMP and LFD was used to detect the catfish pathogen *E. ictaluri*. For this assay, the LAMP reaction was formulated and performed using the standard protocol except to facilitate LF detection, forward and reverse loop primers were labeled as described in the methods section. Results presented in Figure 10 shows sensitivity and specificity of *E. ictaluri* LAMP as determined using LFD. Appearance of a red line only at “Control” indicated that no product was amplified/or detected in any of the dilutions from both *E. coli* and *E. tarda* cell cultures, confirming 100% specificity of *E. ictaluri* LAMP (Figure 10; lower panel).

In contrast, product was amplified and detected from the cell cultures of both *E. ictaluri* strains as well as positive controls indicating presence of target gene in the samples (Figure 10, upper panel). Positive reaction was detected only in dilutions −2, −4, and −6 indicating sensitivity of *E. ictaluri* LAMP equivalent to approximately 8 cells (starting concentration = 10^9^ CFU mL^−1^).

## Discussion

Nucleic acid tests (NATs) offer major advantages in terms of speed and sensitivity for pathogen detection, but these assays are not simple or inexpensive enough to implement in resource-limited settings. However, development of LAMP technology has changed this paradigm and has given new impetus toward diagnostic methods suitable for use without extensive training or equipment. LAMP (Notomi et al., 2000; Mori and Notomi, 2009; Mori et al., 2013) is a nucleic acid amplification method that is highly amenable to isothermal detection and best suited to overcome some of the disadvantages of other NATs (PCR, real time PCR). This method uses four or more primers, two of which are engineered to generate loop structures in the nascent strand that primes a cascade of DNA synthesis resulting in microgram yields of amplification product from as low as single-copy targets in as little as 10 min. Well-designed LAMP tests rival real-time PCR in sensitivity and specificity and excel in simplicity of set-up and time to result.

A POC molecular diagnostic test using LAMP based assays is readily achievable, as the only instrument requirement is an inexpensive heater. Portable, battery operated heaters can be improvised (Hernandez et al., 2011) for remote detection amenable to use by individuals with very little training. In some cases, these assays are miniaturized and coupled to hand held devices which would allow instantaneous reporting of results to a central database from virtually any corner of the planet (Stedtfeld et al., 2012; Myers et al., 2013). In other cases, field operation is facilitated by detection of the amplification product using an inexpensive lateral flow device that provides an unambiguous easily interpreted result (Ge et al., 2013). During the last 10 years, LAMP based methods have been developed for detection of various pathogens (Parida et al., 2008; Fu et al., 2011; Mori et al., 2013). Conventionally, LAMP uses *Bst* polymerase for amplification of DNA targets. In this paper, we report on applications and advantages of using OmniAmp polymerase in DNA and RT-LAMP reactions.

OmniAmp Pol has a unique combination of properties, including strand displacement, thermostability and reverse transcriptase activity that make it uniquely suitable for use in LAMP formulations for detection of both DNA and RNA targets without modification of the buffer formulation or work flow. In this study, we showed the ability of a single formulation of OmniAmp polymerase to amplify 4 bacterial and viral DNA targets such as *E. ictaluri, S. aureus*, *B. atrophaeus*, and PCV-2; and 6 RNA viral targets such as WNV, CCHF, EBoV, SIV, MS2, and BVDV. All of the targets amplified in under 30 min with high sensitivity and no alteration of formulation or process. In comparison, RT-LAMP using *Bst* polymerase requires pre-incubation with a reverse transcriptase, typically AMV RT for detection of RNA targets (Notomi et al., 2000; Tanner and Evans, 2014).

Post-incubation, separation of reaction products on 2% agarose gel showed ladder like patterns, which is typical of LAMP (Notomi et al., 2000). In certain cases, where non-specific amplifciation was observed, melt analysis was used to differentiate between specific and non-specific products. Yamamura et al. (2009) has shown the utility of melt analysis in enabling identification of correct amplification products.

The thermostability of OmniAmp polymerase compared to *Bst* polymerase translates into faster TTR, particularly with more dilute templates. Thermostability is especially important for amplification of GC rich targets or those with extensive secondary structure as high temperature incubation can be used to relax secondary structure. We showed utility of this approach in LAMP method for BVDV. Design parameters for bovine viral diarrhea virus (BVDV) type I LAMP primers is highly constrained by the overall variability of the BVDV genome (Deng and Brock, 1993). This variability limits primer designs to the conserved 5′-UTR, which is highly structured. However, brief incubation at 92°C for 3 s before isothermal incubation enabled amplification through the secondary structure in the 5′-UTR region. In contrast, *Bst* polymerase is not stable above approximately 68°C, and cannot be used for high temperature denaturation of structured targets.

Having a simple and easy to use sample preparation method is one of the major criteria for a true POC diagnostic test. Toward this end, we developed a simple heat lysis method for extraction of nucleic acid and crude lysates used as template in LAMP reaction. No inhibitory effects were observed, indicating that performance of OmniAmp Pol is not impacted by presence of sample matrix components that act as contaminants in PCR based amplification. Another major unmet requirement for POC diagnostics in resource limited settings is long shelf life without maintaining refrigeration or other means of a cold chain (Mabey et al., 2004; Nijru, 2012). The dried formulations described in this report were stable at ambient temperature (23°C) and 37°C for at least 6 months with no apparent loss in activity.

Positive LAMP reactions can be detected by agarose gel electrophoresis or spectrophotometric measurement of turbidity; however, these methods are not amenable to POC use. Fluorescent detection using dyes such as calcein (Tomita et al., 2008), or HNB (Goto et al., 2009), offers easy to use detection methods. Because these dyes can bind to any dsDNA, they fail to distinguish between specific and non-specific amplification products (Nijru, 2012; Ge et al., 2013). Use of SYBR green I is also not suitable for field applications as it has to be added after the completion of the reaction, a step which increases risk of contamination (Nijru, 2012). LFD has been explored as a means of detecting positive LAMP reactions (Njiru, 2011; Ge et al., 2013; Roskos et al., 2013). In this study, we evaluated LFD in combination with OmniAmp polymerase-based LAMP to visualize amplification products. This method improves specificity due to the secondary binding and detection of amplicon specific targets and negates the need for techniques and instruments unavailable in many low resource settings. The method of labeling the loop primers with biotin and FITC was found to provide high sensitivity and specificity for detection of true positive amplification products. In the present study, we could detect as little as eight cells from two different strains of *E. ictaluri* with no amplification of non-specific targets (*E. tarda and E. coli*). These results suggest high sensitivity and specificity of the detection method (LAMP coupled with LF) and shows utility of LFD as a simple and easy to use read out method for visualization of LAMP results.

## Conclusion

Results presented in this paper show the utility of OmniAmp polymerase in LAMP assays for detecting both RNA and DNA targets. This formulation provides advantages in sample preparation, speed, shelf-stability, and reliability on structured templates compared to traditional LAMP enzymes. We also provide a POC compatible means of detecting positive reactions using LFD.

## Author contributors

Yogesh Chander helped conceive project, designed and performed experiments and wrote the manuscript. Jim Koelbl, Michael J. Moser, Audrey J. Klingele, Abel Carrias, and Jamie Puckett designed and executed experiments. Mark R. Liles conceived and interpreted experiments. David A. Mead helped conceive project and edit the manuscript. Thomas W. Schoenfeld conceived the project and edited the manuscript.

### Conflict of interest statement

Yogesh Chander, Jim Koelbel, Jamie Puckett, Michael Moser, Audrey Klingele, David Mead, and Thomas Schoenfeld are employed by Lucigen Corporation. Lucigen has commercialized the OmniAmp polymerase for research use only. Mark Liles and Abel Carrias have no commercial or financial relationship with Lucigen Corporation, WI and declare no conflict of interest.
